# Desmoplastic small round cell tumour: characteristics and prognostic factors of 41 patients and review of the literature

**DOI:** 10.1186/2045-3329-3-14

**Published:** 2013-11-26

**Authors:** Han Hsi Wong, Helen M Hatcher, Charlotte Benson, Omar Al-Muderis, Gail Horan, Cyril Fisher, Helena M Earl, Ian Judson

**Affiliations:** 1 Addenbrooke’s Hospital, Cambridge University Hospitals NHS Foundation Trust, Hills Road, Cambridge CB2 0QQ, UK; 2 Sarcoma Unit, The Royal Marsden, Fulham Road, London SW3 6JJ, UK; 3 Department of Histopathology, The Royal Marsden, Fulham Road, London SW3 6JJ, UK; 4 University of Cambridge Department of Oncology and NIHR Cambridge Biomedical Research Centre, Hills Road, Cambridge CB2 0QQ, UK

**Keywords:** Desmoplastic small round cell tumour, Sarcoma, Prognosis, Survival, Treatment

## Abstract

**Background:**

Desmoplastic small round cell tumour (DSRCT) is a rare but frequently fatal sarcoma, and many of its characteristics still require further clarification.

**Methods:**

We retrospectively analysed 41 patients treated at or referred to two regional referral centres in the UK between 1991 and 2012. A review of the current literature was also performed.

**Results:**

The median age of presentation was 27 years (range 16 to 45 years), with a male-to-female ratio of 3:1. Ninety percent of patients had disease in the abdomen. The median size of the presenting tumour was 13 cm (range 3.5 to 23 cm), and 80% had metastatic disease at diagnosis, mainly in the liver (33%) and lungs (21%). Time-to-progression (TTP) was 3.9, 2.3 and 1.1 months after first-, second- and third-line chemotherapy, respectively. First-line treatment with VIDE chemotherapy appeared to confer the longest TTP (median 14.6 months). Ifosfamide and doxorubicin resulted in TTP of >3.8 months when used in any-line setting. Eleven patients received targeted agents as part of a clinical trial. After a median follow-up of 14 months, the overall median survival (MS) was 16 months. There was no difference in MS with regards to age, gender, or size of the presenting tumour. Patients with extra-abdominal disease survived longer compared to those with tumours in the abdomen (all still alive vs MS of 15 months; *P* = 0.0246). Patients with non-metastatic intra-abdominal disease who underwent surgery had an MS of 47 months (16 months for those who did not have surgery; *P* = 0.0235). Radiotherapy for locoregional control in patients with metastatic intra-abdominal DSRCT was associated with longer survival (MS of 47 vs 14 months; *P* = 0.0147).

**Conclusions:**

DSRCT is a rare but often fatal disease that mainly affects younger male patients. Those with intra-abdominal DSRCT have a poorer prognosis, although surgical resection for localised disease and radiotherapy in the metastatic setting are associated with improved survival. A patient’s age, gender and size of presenting tumour do not have prognostic significance.

## Background

Desmoplastic small round cell tumour (DSRCT) is a rare but highly aggressive neoplasm that typically occurs in adolescent and young males. An earlier study has found a male-to-female ratio of approximately 5 to 1 and a mean age at diagnosis of 22 years [1]. First described in 1989, it is characterised by clusters of poorly differentiated small round blue cells lying within an abundant fibrosclerotic stroma [2]. These cells co-express epithelial, mesenchymal, myogenic and neural markers [3], but are distinguished by the chromosomal translocation t(11;22)(p13;q12) resulting in the fusion of the Ewing’s sarcoma (*EWSR1*) and the Wilms’ tumour (*WT1*) genes [4]. DSRCT generally develops in the abdomen and have a tendency towards peritoneal spread, with subsequent metastasis to distant lymph nodes, liver and lungs [5].

In this study, we retrospectively reviewed 41 patients with DSRCT who were treated at or referred to two regional sarcoma centres in the UK. Analysis of survival and prognostic factors, as well as review of the current literature on the management of DSCRT, was performed. This series is comparable in size with other large series previously reported.

## Methods

### Patient groups

The Royal Marsden Hospital in London and in Sutton, UK, and Addenbrooke’s Hospital in Cambridge, UK, are regional sarcoma referral centres for London and South East England, and for East of England, respectively. Patients with DSRCT treated at or referred to these two centres between the years of 1991 and 2012 were identified retrospectively. Diagnosis was confirmed by immunohistochemistry and reviewed by central specialist histopathologists. Cytogenetic analysis for *EWSR1-WT1* rearrangement was performed when available. Patients’ medical and treatment records were analysed. All 41 patients identified were included in the study. Approval from the local research ethics committee was obtained prior to data collection.

### Statistical analysis

Time-to-progression (TTP) is the time interval from completion of chemotherapy to radiological disease progression as defined by Response Evaluation Criteria in Solid Tumours (RECIST). Survival analysis was performed using the Kaplan-Meier method and log rank test. Surviving patients were censored at last contact.

## Results

### Patient characteristics

A total of 41 patients were referred to or treated at the centres between 1991 and 2012. Thirteen of these were referred for opinion only and were managed subsequently at their local hospitals. Diagnosis of DSRCT was confirmed by central specialist histopathological review. Cytogenetic testing for *EWSR1-WT1* rearrangement was only routinely available in some centres after 2008, therefore only 14 patients were tested and found to be positive. Patient characteristics are summarised in Table 1. The age of presentation ranges from 16 to 45 years, with a median of 27 years. About three quarter of the patients were males. The majority of patients presented with abdominal or pelvic tumours (i.e. arising from the retroperitoneum or within the peritoneal cavity with no clear indication of organ of origin), with sizes ranging from 3.5 to 23 cm. Four patients with extra-abdominal disease had disease in the prostate, testis, shoulder and thigh, respectively. Eighty percent of the patients had evidence of metastasis at presentation, with lungs and liver being the commonest sites.

**Table 1:** Patient characteristics (n = 41)

**Variables**	**No. of patients (%)**
**Gender**	
Male	31 (76%)
Female	10 (24%)
**Median age (years)**	27 (range 16 – 45)
**Presenting site**	
Abdomen and pelvis	37 (90%)
Prostate	1 (2.4%)
Testis	1 (2.4%)
Shoulder	1 (2.4%)
Thigh	1 (2.4%)
**Median tumour size at diagnosis (cm)**	13 (range 3.5 – 23)
**Metastasis at presentation**	33 (80%)
**Sites of metastasis at presentation (n = 33)**	
Liver	11 (33%)
Lung	7 (21%)
Peritoneal cavity	6 (18%)
Lymph node	5 (15%)
Bone	3 (9%)
Adrenal	1 (3%)
Chest wall	1 (3%)
Prostate	1 (3%)

### Treatments

The treatments received by this cohort of patients are summarised in Table 2. Thirty eight patients (93%) have had chemotherapy, with the majority of them receiving it with palliative intent. Four and two patients received neoadjuvant and adjuvant chemotherapy, respectively. Of these, three subsequently developed metastatic disease. The commonest chemotherapeutic regimes were those frequently used in other small round cell tumours, i.e. a combination of an anthracycline, alkylating agent and vinca alkaloid. Topoisomerase inhibitors, taxanes and platinums have also been used. As the effectiveness of second- or subsequent-line chemotherapy is also unproven, a number of newer agents had been given as part of a clinical trial, including inhibitor of the mammalian target of rapamycin (mTOR) pathway, tyrosine kinase inhibitors (TKIs), and antibody against the insulin-like growth factor-1 receptor (IGF-1R).

**Table 2:** Treatment and chemotherapeutic regimens (n = 41)

**Variables**	**No. of patients (%)**
**Treatment modality**	
Chemotherapy	38 (93%)
- Neoadjuvant	4 (10%)
- Adjuvant	2 (5%)
- Palliative (including relapse after neoadjuvant/ adjuvant treatment)	35 (85%)
Radiotherapy	
- Radical	2 (5%)
- Palliative	4 (10%)
Surgery and its indications	
- Diagnosis	14 (34%)
- Resection/optimal debulking of primary tumour	8 (20%)
**Chemotherapy**	
First-line	
- Vincristine + ifosfamide + doxorubicin + etoposide (VIDE)	13
- Ifosfamide + vincristine + actinomycin D + doxorubicin (IVADo)	7
- Cyclophosphamide or ifosfamide + etoposide	4
- Vincristine + doxorubicin + cyclophosphamide (VAC) or vincristine + ifosfamide + doxorubicin	4
- Bleomycin + etoposide + cisplatin	2
- Ifosfamide + doxorubicin	2
- Carboplatin + etoposide	1
- Carboplatin + paclitaxel	1
- Cisplatin + doxorubicin/paclitaxel + cisplatin + ifosfamide	1
- Doxorubicin	1
- Epirubicin + cisplatin + capecitabine	1
- Ifosfamide + carboplatin + etoposide	1
- Ifosfamide + vincristine + actinomycin D	1
Subsequent-line (with chemotherapeutic agents)	
- Platinum + etoposide	10
- Etoposide	5
- Ifosfamide + etoposide	5
- Doxorubicin	2
- Ifosfamide + doxorubicin	2
- Cyclophosphamide + topotecan	2
- VAC	2
- Gemcitabine or irinotecan + temozolomide	2
- Albumin-bound paclitaxel	1
- Cisplatin + mitomycin C + irinotecan	1
- Doxorubicin + etoposide	1
- IVADo	1
- Liposomal doxorubicin	1
- VIDE	1
Subsequent-line (with non-standard therapies)	
- Figitumumab	2
- Sirolimus + cyclophosphamide or liposomal doxorubicin	2
- Pazopanib +/− etoposide	2
- Sunitinib	2
- Axitinib	1
- Goserelin	1
- Interferon	1
- Semaxanib	1
- Sorafenib	1

In our series, only six patients received radiotherapy. One patient each had radical radiotherapy after resection of an abdominal (54 Gy in 30 fractions) and thigh (60 Gy in 30 fractions) tumour, respectively. The remaining four patients received palliative conformal radiotherapy (20 Gy in five fractions) to the abdomen for locoregional control in metastatic disease. Resection or optimal debulking of the primary tumour was done in eight patients, whereas in 14 patients surgery was performed for diagnostic purposes.

### Time-to-progression and survival

TTP after first- to third- line systemic therapies are summarised in Table 3. Unsurprisingly, the median TTP decreases with increasing lines of treatment. First-line treatment with VIDE chemotherapy appeared to confer the longest TTP (median of 14.6 months; range 1.9 to 33.7 months). In second-line treatment, etoposide alone (n = 1), or in combination with platinum (n = 9) or ifosfamide (n =2), were most commonly used, with a median TTP of 3.4 months (range 0.3 to 13.9 months). Ifosfamide and doxorubicin treatment resulted in a median TTP of >3.8 months when used in any line-setting.

**Table 3:** Time-to-progression after systemic therapy

**Line of treatment**	**Median time-to-progression (months)**
First (n = 38)	3.9 (range 0.6 to 33.7)
Second (n = 23)	2.3 (range 0.3 to 13.9)
Third (n = 13)	1.1 (range 0.6 to 11.8)

**Figure 1:**
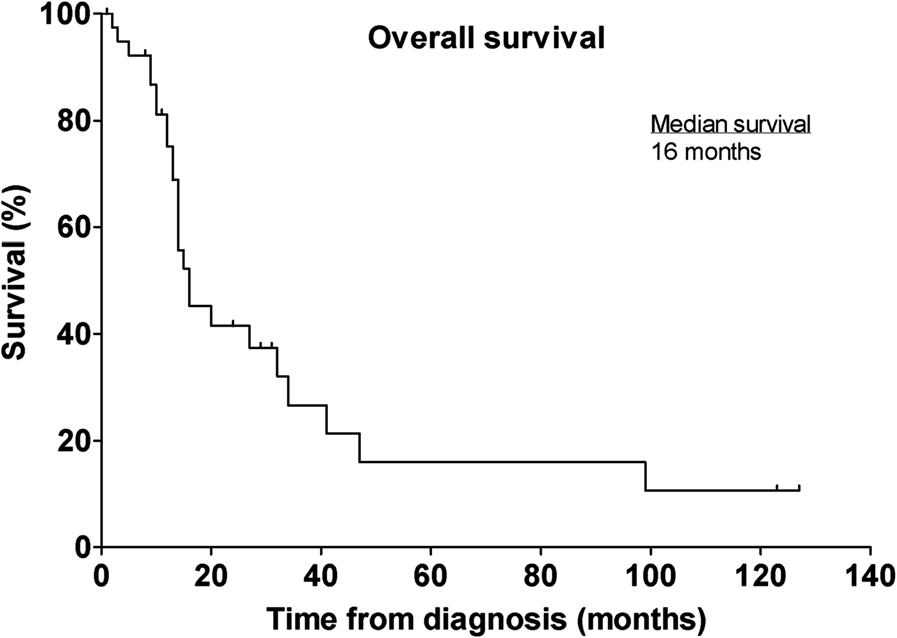
Kaplan-Meier plot of overall survival for the entire cohort (n = 41).

The median follow-up period for all patients was 14 months (range 1 to 127 months). Sixteen (39%) patients were still alive at a median follow-up of 12.5 months. All deaths were due to the disease. The overall median survival (MS) was 16 months (range 2 to >127 months) (Figure 1). Three-year and 5-year survival rates were 27% and 16%, respectively. The longest surviving patient had disease in his prostate, and he presented early with symptoms of urinary outflow obstruction. He still has no evidence of disease for more than 10 years after his initial diagnosis. Three other patients who presented early with lumps in the testis, shoulder and thigh, respective, are still alive at the time of last follow-up, albeit with evidence of metastatic disease.

**Figure 2:**
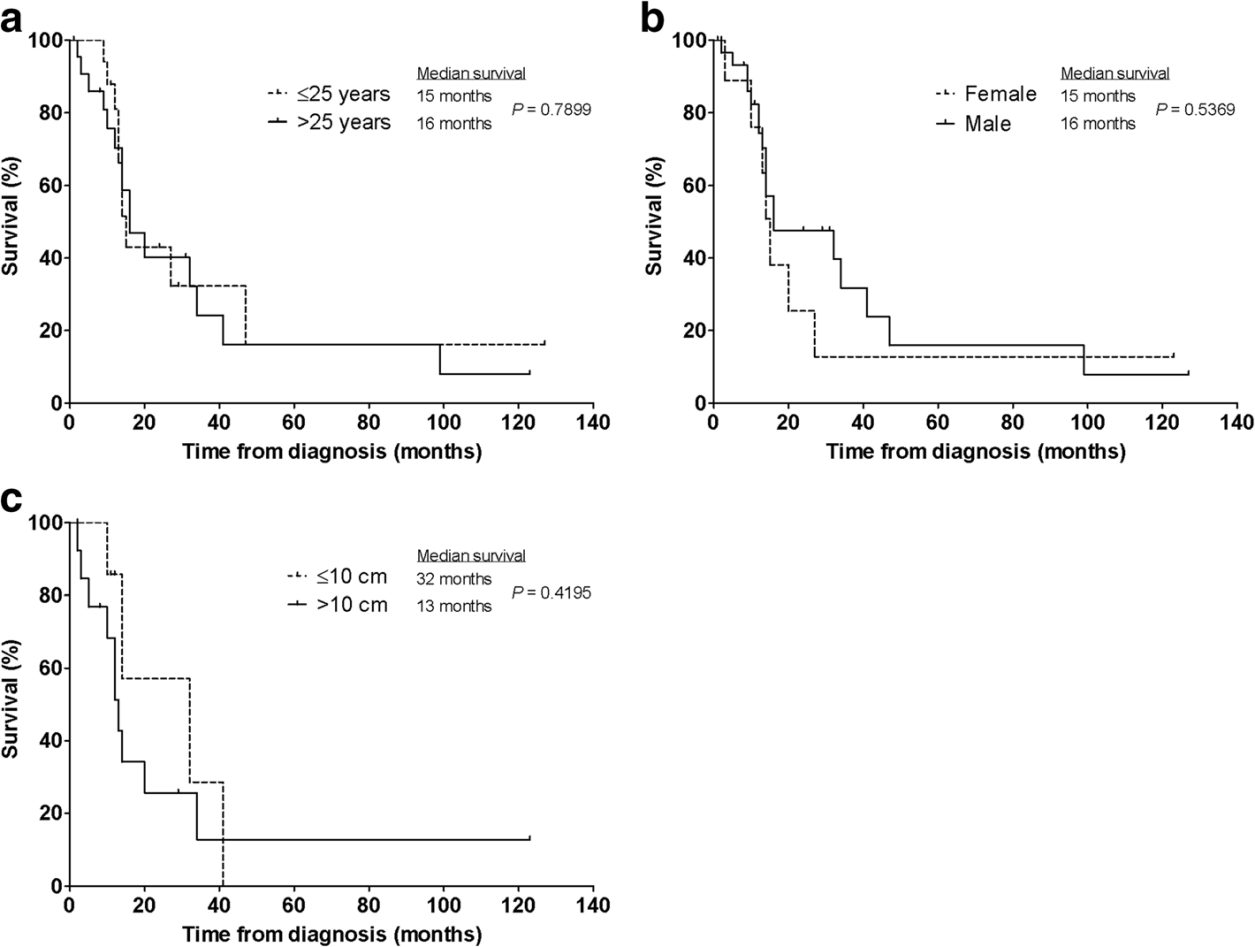
**Factors not associated with prognostic significance.** Kaplan-Meier plots of overall survival stratified according to patient’s **(a)** age, **(b)** gender and **(c)** size of the primary tumour.

There was no difference in MS with regards to age (15 vs 16 months for those ≤25 years and >25 years, respectively; *P* = 0.7899), gender (16 vs 15 months for males and females, respectively; *P* = 0.5369), or whether the presenting tumour was ≤ or >10 cm (32 vs 13 months; *P* = 0.4195) (Figure 2).

Patients with extra-abdominal disease (n = 4) survived longer compared to those with tumours in the abdomen or pelvis (n = 37) (all still alive vs MS of 15 months, respectively; *P* = 0.0246). Patients with non-metastatic, intra-abdominal and -pelvic disease at presentation who had undergone surgical resection of the primary tumour (n = 6) survived much longer than those who did not have surgery (n = 11) (MS of 47 vs 16 months, respectively; *P* = 0.0235). The decision on surgery depended largely on the site of disease and resectability. Four patients who underwent resection had received either neoadjuvant (n = 2) and adjuvant (n = 2) chemotherapy, one of whom remains disease-free 10 years after his curative surgery.

**Figure 3:**
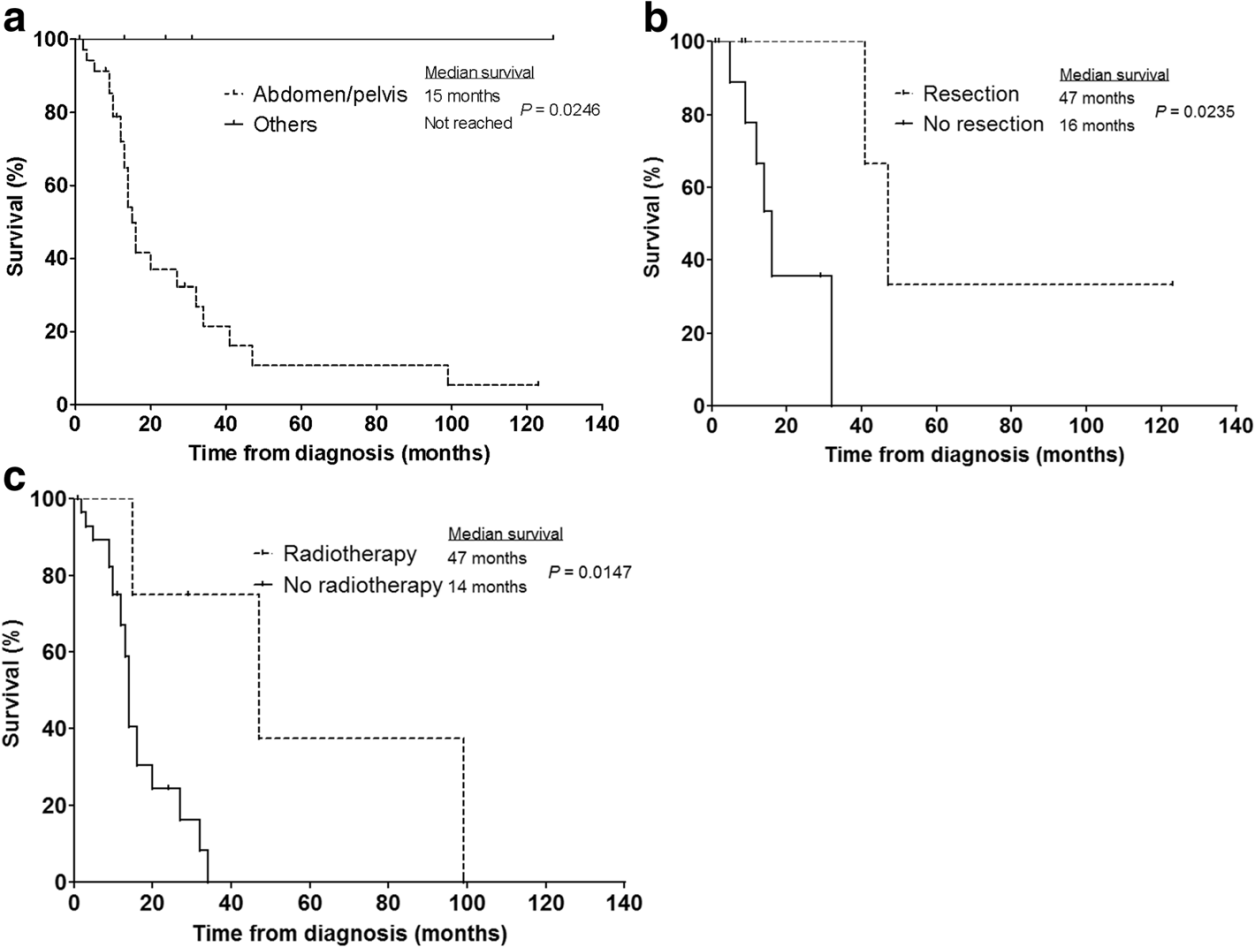
**Factors associated with prognostic significance.** Kaplan-Meier plots of overall survival stratified according to **(a)** site of disease (abdomen/pelvis (n = 37) or other sites (n = 4)), **(b)** whether patients had undergone surgical resection (n = 6) or not (n = 11) for localised intra-abdominal disease and **(c)** whether patients with intra-abdominal metastatic disease had radiotherapy (n = 4) or not (n = 29) for locoregional control.

Four patients with metastatic, intra-abdominal DSRCT had radiotherapy for locoregional control – when compared to a similar group of patients who did not receive radiotherapy (n = 29), a significant difference in MS was noted (47 vs 14 months, respectively; *P* = 0.0147) (Figure 3).

## Discussion and review of the literature

In this report, we present one of the largest series of patients with DSRCT. Consistent with previously published data, DSRCT tends to occur in younger males. Most patients presented with abdominal or pelvic tumours and many have evidence of metastases, the majority being in the lungs and liver.

### Multimodality treatment of DSRCT and prognostic factors

The reported MS of DSRCT is in the region of 17 to 25 months [6]. Given the poor outcome of the disease and the significant morbidities and mortality associated with its treatment, prognostic indicators are very important. Two retrospective studies performed at the Memorial Sloan-Kettering Cancer Center (MSKCC) have both found that aggressive surgical debulking of DSRCT is of prognostic significance [7,8]. An analysis of 32 patients by Schwarz *et al*. demonstrated that improved survival was found to be associated with the following: more than 90% surgical debulking either before or after chemotherapy, complete or very good partial response (PR) to multimodality treatment, and use of the P6 protocol (see below) [7]. In a report of 66 patients by Lal *et al*., treatment with chemotherapy, surgery and radiotherapy conferred a 3-year survival of 55% compared to 27% for those who did not receive all three treatments [8]. In addition, gross tumour resection was also associated with prolonged survival (3-year survival of 58% compared to 0% in the non-resected patients). Naturally such an analysis performed retrospectively cannot adequately control for the fact that patients with radically resectable disease are likely to have less bulky and more localised tumours.

There is no general consensus on the best therapeutic approach, as strong evidence is lacking given the rarity of the disease, although multimodality treatment with chemotherapy, surgery and radiotherapy appears to represent optimal management. The MS of patients diagnosed with DSRCT was 16 months in this study, which is slightly lower than those reported previously [6]. Comparatively, it is clear that the management in our centres took a more conservative approach than others, as evident by the less frequent use of radiotherapy, surgery and myeloablative chemotherapy with stem cell transplantation. In a review by Hassan *et al*. of 12 patients with intra-abdominal DSRCT (all of whom had received multi-agent chemotherapy), those who underwent surgical resection had a longer MS of 34 months compared to 14 months for those who had biopsy alone [9]. In our study, the MS observed for patients who had resection for their abdominal or pelvic tumours was 47 months, compared to 16 months for those who did not. Moreover, for patients with metastatic intra-abdominal DSRCT, palliative radiotherapy for locoregional disease control appeared to confer a survival advantage (MS of 47 vs 14 months in those who did not have radiotherapy). Although patients with localised abdominal or pelvic disease who underwent surgery appear to have similar MS (i.e. 47 months) compared to those with metastatic disease who received palliative radiotherapy, they are by no means comparable and surgery is still indicated in resectable DSRCT. In our series, the only patient with abdominal disease who has been cured (disease-free 10 years from diagnosis) has had chemotherapy and surgical resection. Hence, a more aggressive multimodality treatment approach would seem to be indicated in order to prolong survival, although larger prospective trials with quality-of-life measures would be necessary to confirm this. This is difficult to perform in such a rare disease.

Subbiah *et al*. presented the largest series of patients diagnosed with DSRCT at the 2012 American Society of Clinical Oncology meeting [10]. This was a retrospective review of 197 patients treated at the MD Anderson Cancer Center (MDACC) and MSKCC. In that series, 87% were males with a mean age of presentation of 25 years. In total, 139 (71%) patients underwent surgery, 38 (19%) had debulking surgery, 30 (15%) received radiotherapy, 27 (14%) had intraperitoneal chemotherapy after debulking, and 11 (5%) had stem cell transplant. They found that radiotherapy, surgery, intraperitoneal chemotherapy, removal of primary mass and metastases, age <30 years and patients treated after 2003 were associated with improved survival. In contrast, our study did not show a difference in survival with regards to age of presentation. We also found that the patient’s gender and size of the presenting tumours do not have an impact on survival. Although uncommon, patients who did not have disease in the abdomen appeared to have a better outcome. This is likely to be related to earlier presentation, less advanced disease and in some cases the feasibility of radical resection.

### Chemotherapy

DSRCT is sensitive to chemotherapy although a transient response followed by disease progression is the norm. Chemotherapeutic regimes used are normally similar to those for treating Ewing’s sarcoma. Farhat *et al*. treated five patients with a chemotherapeutic regime consisted of cisplatin, etoposide, cyclophosphamide and an anthracycline – disease stabilisation lasting 4 to 9 months were noted in four patients with intra-abdominal DSRCT after initial surgery, whereas one patient with relapsed metastatic disease from an initial paratesticular primary attained a complete response (CR) [11]. The authors also reviewed the literature of 60 patients who were treated by chemotherapy with or without abdominal radiotherapy, and objective responses were found in 17 patients, eight of whom achieved a CR. The chemotherapy agents associated with CR were those of doxorubicin, cyclophosphamide, vincristine and cisplatin. These are in line with our results, with these drugs providing the longest TTP. Chemotherapy is often given in the metastatic setting; and although often used, the role of neoadjuvant and adjuvant chemotherapy in localised disease remains unknown.

Kushner *et al*. reported 12 patients (10 treatment-naïve and two had previous chemotherapy) who received the P6 protocol, which has seven courses of chemotherapy consisting of cyclophosphamide, doxorubicin, vincristine (HD-CAV), etoposide and ifosfamide [12]. This was followed by surgery, radiotherapy, and myeloablative chemotherapy using thiotepa and carboplatin with stem cell rescue in some cases. All tumours showed a PR with this regimen although there was no CR, and survival of around 20 months was reported. This protocol is used in many centres, mainly in resectable cases, although treatment-related toxicities could be severe. Whether the intensive P6 regimen is better than standard first-line chemotherapy regimens used in other small round blue cell tumours, such as Ewing’s sarcoma, is unknown. In two prospective studies by Bertuzzi *et al*., a total of 17 patients were treated with induction chemotherapy consisting of ifosfamide, epirubicin and vincristine – those who responded were then treated with high-dose chemotherapy and stem cell rescue in conjunction with local therapy (surgery and/or radiotherapy) [13,14]. Approximately half of them achieved an initial PR to induction chemotherapy, but no CR was achieved with high-dose chemotherapy. The MS reported was 14 months, leading the authors to question the role of high-dose chemotherapy in the treatment of DSRCT. More recently in a retrospective study using data obtained from the Center for International Blood and Marrow Transplant Research, Cook *et al*. reported the outcome of 36 DSRCT patients who had undergone autologous stem cell transplantation [15]. The benefit was much greater for those who achieved a CR pre-transplantation compared to those who did not, with MS of 36 and 21 months, respectively.

The use of other chemotherapy drugs has been reported, including irinotecan, temozolomide and vinorelbine, but none of them showed superiority [16,17]. Evidence and experience is emerging on the role of trabectedin in the management of metastatic DSRCT [18-20]. In a case report, an 18-year-old boy with abdominal DSRCT was initially treated by complete surgical excision, followed by adjuvant chemotherapy with cyclophosphamide, doxorubicin/actinomycin D, vincristine, alternating with ifosfamide and etoposide [20]. Disease recurrence was treated with surgery and cisplatin and irinotecan, but this was followed by further progression for which trabectedin was given, resulting in PR. This resulted in a survival of 4 years from diagnosis.

Similar to ovarian cancer, the use of hyperthermic intraperitoneal chemotherapy has also been reported given the tendency of the disease to spread within the peritoneum. Heated cisplatin is given at a dose 100 to 150 mg/m^2^ intraperitoneally after optimal cytoreductive surgery. The series reported by the MDACC showed that this method is safe and might have activity in paediatric patients, and a survival benefit has also been reported [10,21-23]. Further studies are required before this could be widely adopted.

### Radiotherapy

Whole-abdominopelvic radiotherapy (WAP-RT) after maximal surgery was first reported by Kushner *et al*. at the MSKCC as part of a multimodality treatment using the P6 protocol, with the aim of improving local control [12]. A total dose of 30 Gy was delivered post-operatively, with simultaneous boost given to sites of gross residual disease. Conventional two-dimensional radiotherapy was associated with significant gastrointestinal and haematologic toxicities, with long term side effects including small bowel obstruction and ureteral stenosis [24]. For this reason, the use of WAP intensity-modulated radiation therapy (WAP-IMRT) was studied by Pinnix *et al*. at the MDACC [25]. All of the eight patients had received prior chemotherapy and surgical debulking (seven of them also had intraperitoneal cisplatin). One patient was still disease-free 20 months after treatment, although the rest experienced either local or distant failure after a median of 8.73 months from WAP-IMRT. A retrospective analysis at the MSKCC looked at 31 patients who underwent WAP-RT, either with conventional two-dimensional radiotherapy (n = 22) or IMRT (n = 9) after chemotherapy and maximal debulking surgery [26]. IMRT was associated with lower incidence of acute gastrointestinal and haematologic toxicities. The 3-year overall survival and progression-free survival (PFS) rates were 50% and 24%, respectively. Anecdotally, a patient in this series who received WAP-RT developed a serious malabsorption syndrome subsequent to gastrointestinal damage. Given the limited data of efficacy, WAP-RT is currently not routinely used in the management of DSRCT.

### Targeted therapies

In recent years, targeted therapies have been studied in DSRCT. Drugs that have shown activity against this disease include the TKI sunitinib and the mTOR inhibitor temsirolimus [27,28]. In our cohort of patients, other non-standard agents used include the anti-IGF-1R antibody figitumumab, the TKIs axitinib, pazopanib, sorafenib and sunitinib, as well as the mTOR inhibitor sirolimus. The number of patients is too small to draw any conclusion about their efficacy. Due to the fact that DSRCT has a predilection to occur in young males, Fine *et al*. discovered that androgen receptor is expressed in 37% of DSRCT [29]. Six of their patients were treated with combined androgen blockade and three attained a clinical benefit. In our study, one patient had received the gonadotropin-releasing hormone agonist goserelin. However, no significant anti-tumoural efficacy was noted.

Chromosomal translocation resulting in the fusion of the *EWSR1* and *WT1* genes is the molecular characteristic of DSRCT. The resulting fusion protein has been found to activate the IGF-1R gene promoter, causing the expression of this anti-apoptotic receptor tyrosine kinase [30-32]. The understanding of this mechanism has provided a novel target for the treatment of this disease. In a recent phase II study, 16 patients with DSRCT who had had previous treatments were given 12 mg/kg of the anti-IGF-1R antibody ganitumab intravenously [33]. Common side effects include fatigue, nausea, dyspnoea and peripheral oedema. PR was noted in one (6%) patient, whereas 10 (63%) had stable disease (SD) as their best response, with 3 (18%) achieving SD lasting ≥24 weeks. Median PFS was 19 months, indicating a potential role of ganitumab used either alone or in combination with chemotherapy for patients with DSRCT. In a phase I study of another anti-IGF-1R antibody cixutumumab in combination with temsirolimus, two out of three patients with previously-treated DSRCT had SD lasting longer than 5 months [34].

Tumour-specific antigens have also been studied as targets for immunotherapy, including the disialoganglioside GD2 and the antigen recognised by the antibody 8H9 (expressed in 70% and 96% of DSRCT, respectively) [35]. In particular, studies of anti-GD2 antibodies have shown some promising results in the treatment of neuroblastoma [36]. Another potential therapeutic target is the lysine-specific demethylase 1, a key histone modification enzyme involved in controlling gene expression which if dysregulated, could result in tumourigenesis [37]. It is found to be highly expressed in several highly malignant sarcomas including DSRCT [38]. It could be inhibited by small molecule inhibitors and further investigation is warranted.

## Conclusions

Advanced DSRCT is a rare, aggressive disease with invariably poor outcome that generally occurs in young men. It has a propensity to metastasise and at present, surgery, combination cytotoxic chemotherapy and radiotherapy remain the only standard therapeutic options. In our study, we found that patients with intra-abdominal DSRCT have a poorer prognosis, although surgical resection for localised disease and radiotherapy, even in the metastatic setting for locoregional control, are associated with improved survival.

Clearly more efforts are needed to improve the prognosis of patients with DSRCT, and the development of novel targeted agents is likely to have a major role in altering the course of the disease. It is also hope that the International Rare Cancers Initiative, a multinational collaboration with the aim of developing clinical trials for uncommon malignancies, will help to address this issue in the future.

## Competing interests

The authors declare that they have no competing interests.

## Authors’ contributions

All authors were involved in data collection. HHW, HMH and OA analysed the data. HHW and HMH drafted the manuscript. HHW, HMH, CB, GH, CF, HME and IJ all contributed to the manuscript preparation. All authors read and approved the final manuscript.
